# Tc 99m bone scan and fluorodeoxyglucose positron emission tomography in evaluation of disseminated langerhans cell histiocytosis

**DOI:** 10.4103/0972-3919.78253

**Published:** 2010

**Authors:** Sait Sager, Sabire Yilmaz, Gunes Sager, Metin Halac

**Affiliations:** Department of Nuclear Medicine, Istanbul University, Cerrahpasa Medical Faculty, Istanbul, Turkey; 1 Goztepe Education and Research Hospital, Clinic of Pediatrics, Istanbul, Turkey

**Keywords:** Bone scan, FDG PET/CT, langerhans cell histiocytosis, Tc-99m MDP

## Abstract

Langerhans cell histiocytosis (LCH) is a rare histiocytic disorder in which pathological langerhans cells accumulate in a variety of organs. Manifestations may include lung infiltrates, lymph node involvements, bone lesions, hepatic, hematopoietic and endocrine dysfunctions. In this case report we present fluorine-18 positron emission tomography (F-18 PET/CT) and bone scintigraphy findings of a 18-year-old male patient with disseminated LCH, mimicking multiple hypermetabolic metastatic lesions. Clinicians should be aware that LCH infiltrations can be seen as intense uptake and to differentiate infiltrations from other metastatic intense uptake with fluorodeoxyglucose PET/CT and bone scintigraphy, clinical and laboratory findings should be kept in mind.

## INTRODUCTION

Langerhans cell histiocytosis (LCH) is characterized by a proliferation of abnormal dendritic mononuclear cells, known as langerhans cells, with infiltration into multiple organs to systems locally or diffusely.[1] This disease can be presented as a localized or disseminated form. The most common sites of involvement are skin, bone, lymph nodes, lungs, liver and central nervous system. LCH affects mostly at early ages of life and rare in adult age groups. Males are affected more frequently than females.[2]

Clinical manifestations of LCH can range from asymptomic lesions to significant morbidity depending on the kind and number of the organs involved. It can cause distinct clinical syndromes that have been historically described as eosinophilic granuloma, Hand-Schüller-Christian disease, and Letterer-Siwe disease.[3] Multifocal chronic LCH is self-limited in most cases, but increased mortality has been observed among infants with pulmonary involvement.[4] Diagnosis of the disease is based on biopsy.

Imaging of LCH with conventional radiological modalities is diffucult. Because this disease may manifest with a heterogeneous spectrum of lesions, ranging from a single bone lesion to multisystem disease. Bone scintigraphy and direct radiographical imaging have limited use for disseminated LCH. Computed tomography (CT) and magnetic resonance imaging (MRI) methods are effective in evaluation of unifocal skull, vertebral and pelvic lesions.[5]

Fluorine-18 fluorodeoxyglucose positron emission tomography (F-18 FDG-PET) scanning is able to identify LCH in tissues and organs including lymph nodes, spleen and lungs.[6] Functional imaging with FDG-PET is a valuable method for non-invasively detecting active disease.

In this case we present a 18-year-old male patient with disseminated LCH. FDG PET/CT and bone scintigraphy imaging findings showed multiple hypermethabolic lesions mimicking metastatic uptake because of delayed diagnosis.

## CASE REPORT

An 18-year-old male patient was referred to our Nuclear Medicine Clinic for evaluation of bone lesions which were recently seen in thorax CT and also to find other possible lesions with bone scintigraphy and FDG PET/CT imaging. FDG PET/CT was required to find possible primary unknown malign tumor focus.

**Figure 1:**
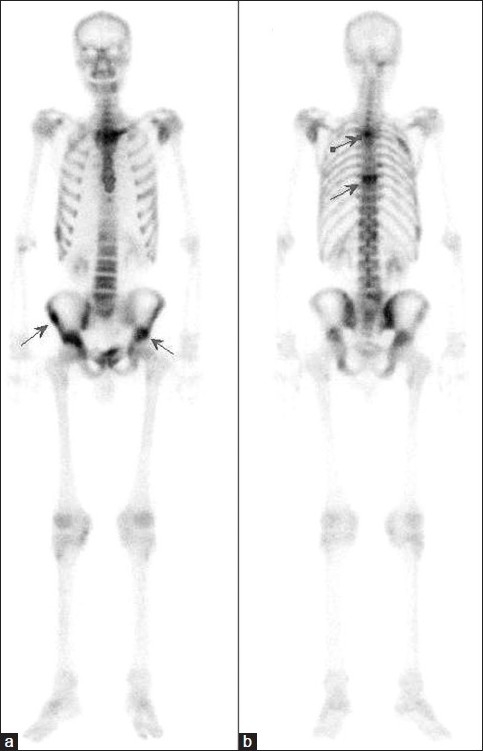
Tc 99m MDP bone scan revealed increased activity in dorsal 2, dorsal 8, right second rib, left scapula, sternum and bilateral iliac bones (a and b, arrows)

A whole body bone scintigraphy with ^99m^Tc methylene diphosphonate (MDP) was performed to evaluate entire skeletal system. Bone scan was obtained using a dual head gamma camera (ESoft ; Siemens Medical Systems, Germany) with a high-resolution, low-energy collimator. Three hours following intravenous administration of 740 MBq (20 mCi) Tc 99m MDP, anterior-posterior whole-body scan was obtained. Bone scan revealed increased uptake in dorsal 2, dorsal 8, right 2. rib, left scapula, sternum and bilateral iliac bones [Figure 1].

**Figure 2:**
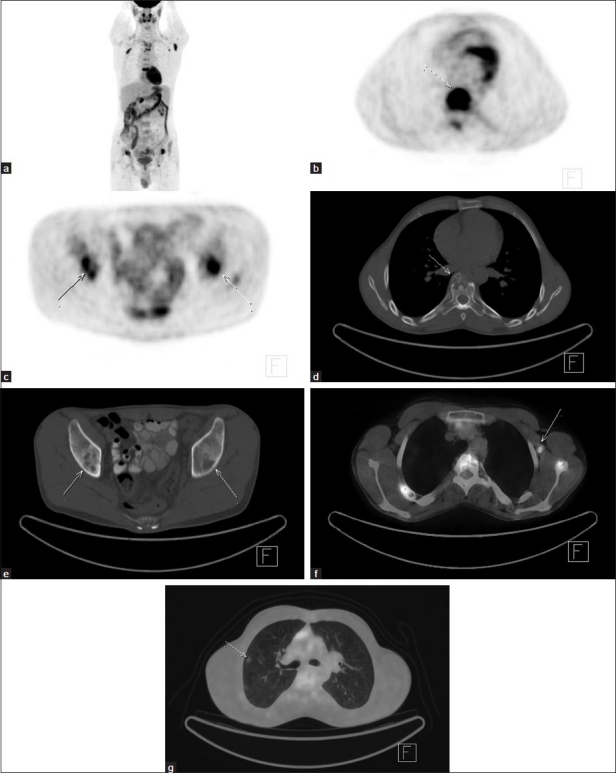
PET/CT imaging revealed intense FDG uptake in dorsal 2, dorsal 8, right second rib, left scapula, sternum and bilateral iliac bones (a, MIP, b and c, axial PET images) which were litic in CT slices (d and e, axial CT images). Maximum standard uptake value of bone lesions was 9,6. In addition to bone lesions FDG uptake in left axillary lymph node with a maxium standard uptake of 5,4 (f, axial fusion image) and minimal FDG uptake in right upper lobe lung pulmonary nodule with a maxium standard uptake of 2,1 were seen (g, axial fusion image)

**Figure 3:**
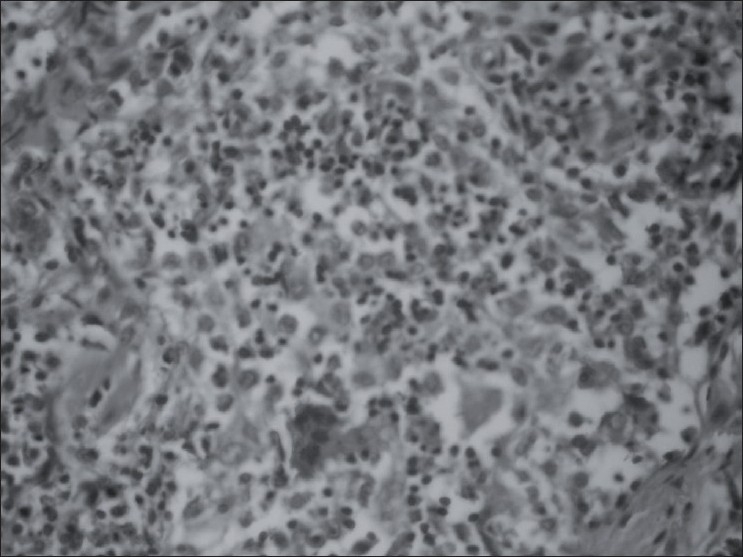
Histological findings of proliferating langerhans cells with intermingled eosinophils, neutrophils and lymphocytes in biopsy specimen (hematoxylin and eosin)

FDG PET/CT examination was performed 2 days after the bone scan. For PET/CT imaging patient was intravenously injected 455 MBq (12,2 mCi) of F18-FDG after 6 hours of fasting period. One hour of waiting time in a silent room patient was imaged using an integrated PET/CT camera, which was consists of a 6-slice CT gantry integrated on a LSO-based fullring PET scanner (Siemens Biograph 6, Chicago, IL, USA). PET/CT imaging revealed intense FDG uptake in dorsal 2, dorsal 8, right 2. rib, left scapula, sternum and bilateral iliac bones which were litic in CT slices [Figure 2]. Maximum standardized uptake value of bone lesions was 9,6. In addition to bone lesions FDG uptake in left axillary lymph node with a maxium standardized uptake of 5,4 and minimal FDG uptake in right upper lobe lung pulmonary nodule with a maxium standardized uptake of 2,1 were seen [Figure 2]. Left axillary lymph node biopsy was recommended to diagnose the primary disease. Biopsy result revealed LCH [Figure 3].

## DISCUSSION

LCH is a rare disasese, mainly affects children, characterized by localized or widespread proliferation of dendritic cells. LCH may present locally or as a multifocal disease that affects only the skeletal system or other tissues and organs, such as lungs, liver and skin.[7]

FDG PET/CT is a sensitive imaging technique which can be used to evaluate patients with LCH and to identify lesions not found by other imaging modalities. Whole body FDG-PET/CT scan can detect LCH activity with greater accuracy than other imaging modalities in bones and soft tissues.[8] A high accumulation of FDG can be observed in histiocytic, fibroblastic and some neurogenic lesions, regardless of whether they are benign or malignant.[9] In addition to giving additional information about the extent of disease, it is also helpful in the follow-up, evaluation of response and detection of early recurrences.[10] FDG PET scan can be also useful as a guide for the identification of possible biopsy sites. In this case, we showed biopsy site in the left axillary lymph node to find primary disease.

Imaging of LCH with conventional radiological modalities is diffucult because it may manifest with a heterogeneous spectrum of lesions, ranging from a single bone lesion to multisystem disease. CT and MRI methods are effective in analyses of unifocal skull, vertebral and pelvic lesions.[11] It is showed that Tc 99m MDP whole body bone scintigraphy is more sensitive than X-ray radiography in detecting histiocytic lesions in ribs, spine, pelvis and less sensitive in identifying lesions in the skull.[12]

To facilitate diagnosis at an early stage, clinicians should be familiar with the modality for imaging of each organ or system. There are case reports indicating the possible role of PET scan in LCH.[13,14] Krajicek *et al*, showed that PET imaging can not reliably distinguish between the benign inflammatory nodular lesions of pulmonary LCH and malignant lesions.[15] In this case report it is showed that disseminated pattern of LCH may mimic multiple metastatic lesions with FDG PET imaging. To differentiate disseminated disease from metastatic uptake at an early stage, clinical and laboratory findings should be correlated.

In conclusion, this case report suggests that 18F-FDG PET/CT is of interest in LCH and it is helpful for the diagnosis of disseminated atypical cases in addition to other imaging modalitied and it allows a better assessment of the disease.

## References

[1] Kaste SC, Rodriguez-Galindo C, McCarville ME, Shulkin BL (2007). PET-CT in pediatric langerhans cell histiocytosis. Pediatr Radiol.

[2] Glotzbecker MP, Carpentieri DF, Dormans JP (2002). langerhans cell histiocytosis: Clinical presentation, pathogenesis, and treatment from the LCH etiology. Univ Pa Orthop J.

[3] Willman CL (1994). Detection of clonal histiocytes in langerhans cell histiocytosis: Biology and clinical significance. Br J Cancer Suppl.

[4] Windebank K, Nanduri V (2009). langerhans cell histiocytosis. Arch Dis Child.

[5] Pavlik M, Bloom DA, Ozgonenel B, Sarnaik SA (2005). Defining the role of magnetic resonance imaging in unifocal bone lesions of Langerhan’s cell histiocytosis. J Pediatr Hematol Oncol.

[6] Shaffer MP, Walling HW, Stone MS (2005). langerhans cell histiocytosis presenting as blueberry muffin baby. J Am Acad Dermatol.

[7] Malpas SJ (1998). langerhans cell histiocytosis in adults. Hematol Oncol Clin North Am.

[8] Phillips M, Allen C, Gerson P, McClain K (2009). Comparison of FDG-PET scans to conventional radiography and bone scans in management of langerhans cell histiocytosis. Pediatr Blood Cancer.

[9] Aoki J, Endo K, Watanabe H, Shinozaki T, Yanagawa T, Ahmed AR (2003). FDG-PET for evaluating musculoskeletal tumors: A review. J Orthop Sci.

[10] Yaman E, Ozturk B, Erdem O, Gokcora N, Coskun U, Uluoglu O (2008). Histiocytic sarcoma: PET-CT evaluation of a rare entity. Ann Nucl Med.

[11] Saliba I, Sidani K, El Fata F, Arcand P, Quintal MC, Abela A (2008). langerhans’ cell histiocytosis of the temporal bone in children. Int J Pediatr Otorhinolaryngol.

[12] Dogan AS, Conway JJ, Miller JH, Grier D, Bhattathiry MM, Mitchell CS (1996). Detection of bone lesions in langerhans cell histiocytosis: Complementary roles of scintigraphy and conventional radiography. J Pediatr Hematol Oncol.

[13] Blum R, Seymour JF, Hickks RJ (2002). Role of FDG-positron emission tomography scanning in the management of histiocytosis. Leuk Lymphoma.

[14] Binkovitz LA, Olshefski RS, Adler BH (2003). Coincidence FDG-PET in the evaluation of langerhans’ cell histiocytosis: Preliminary findings. Pediatr Radiol.

[15] Krajicek BJ, Ryu JH, Hartman TE, Lowe VJ, Vassallo R (2009). Abnormal fluorodeoxyglucose PET in pulmonary langerhans cell histiocytosis. Chest.

